# Design and study of mine silo drainage method based on fuzzy control and Avoiding Peak Filling Valley strategy

**DOI:** 10.1038/s41598-024-60228-x

**Published:** 2024-04-23

**Authors:** Meng Wang, Jiaxu Kang, Weiwei Liu, Meng Li, Jinshuai Su, Zhongzheng Fang, Xin Li, Liyou Shang, Fan Zhang, Chengbin Guo

**Affiliations:** 1 College of Mining Engineering, Liaoning Technical University, Fuxin, China; 2 Xima Coal Mine of Shenyang Coking Coal Co. Ltd, Shenyang, Liaoning China; 3 Mixlinker Networks (Shenzhen) Inc, Shenzhen, Guangzhou China

**Keywords:** Mine drainage, Simulation modeling, Fuzzy control, Avoiding Peak Filling Valley strategy, Coal mine safety, Intelligent mine, Engineering, Electrical and electronic engineering

## Abstract

Coal is a non-renewable fossil energy source on which humanity relies heavily, and producing one ton of raw coal requires the discharge of 2–7 tons of mine water from the ground. The huge drainage task increases the cost of coal mining in coal mines significantly, so saving the drainage cost while guaranteeing the safe production of coal mines is a problem that needs to be solved urgently. Most of the fuzzy controllers used in the traditional dynamic planning methods applied to mine drainage are two-dimensional fuzzy controllers with limited control effect, so the traditional two-dimensional fuzzy controllers are improved by introducing the rate of change of gushing water to form a three-dimensional fuzzy controller with three-dimensional control of instantaneous section—water level—rate of change of gushing water, and at the same time, the optimized dynamic planning method is designed by combining the Avoiding Peak Filling Valley strategy and the optimal dy-namic planning method is used in conjunction with the un-optimized dynamic planning method. The optimized dynamic planning method is applied to the same coal mine water silo gushing water experiments; experimental comparison found that the pumping station system before the optimi-zation of the electricity consumed is 52,586 yuan/day, while after the optimization of the electricity consumed is reduced to 41,692 yuan/day, the cost per day consumed compared with the previous reduction of 20.69%, a year can be saved 3,969,730 yuan. Therefore, the mine water bin drainage method based on fuzzy control and Avoiding Peak Filling Valley strategy proposed in this paper can be used as an improvement method of the existing mine drainage method, which can further ex-pand the economic benefits of coal mines and realize safe production while realizing cost savings.

## Introduction

**Figure 1:**
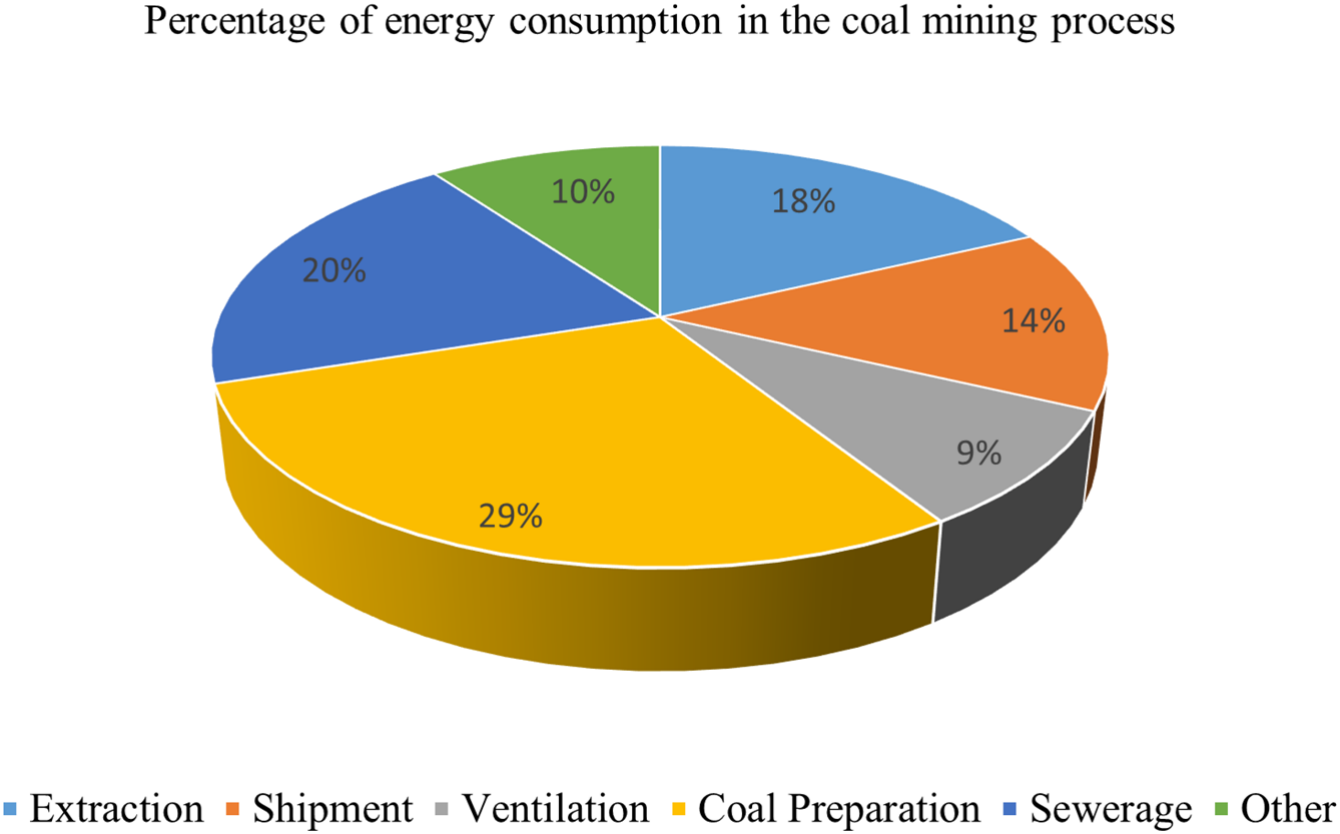
Percentage of energy consumption in the coal mining process.

Coal is an important fossil energy source that human beings rely on for survival. The demand for coal in developing countries and some developed countries is also increasing, so how to realize the safe production of coal mines is a significant issue^1,2^. At the present stage, coal mine safety accidents happen occasionally, among which gas explosion and water damage are the most frequent and severe^3,4^. There are six systems in coal mine production, and the drainage system is one of them^5^. The task of the drainage system is to send the water accumulated in the roadway and working face to the surface^6^. In the process of coal mining, a large amount of mine water will be gathered underground in a coal mine in Henan Province. For example, the production of a ton of raw coal needs to be discharged from the underground 2–7 tons of mine water, the annual output of the coal mine is about 3 million tons, and the mine will produce 6–21 million tons of mine water a year if such a massive amount of mine water can not be discharged in time from the underground, it will produce a significant production safety accidents^7^. Mine drainage systems in the coal production process consume a lot of energy. Its energy consumption will account for about 20% of the total power consumption of the mine, so the design of a reasonable and feasible drainage strategy can significantly reduce the production costs of coal mining enterprises. The energy consumption of the coal production process accounted for such as shown in Fig. 1^8^. Fuzzy control is a nonlinear intelligent control based on fuzzy set theory, fuzzy linguistic variables and fuzzy logic reasoning. This year, a large number of should be in the field of automatic control, the study of fuzzy control as a tool for the realization of the control logic, Avoiding Peak Filling Valley strategy to do the design of the rules of the control process based on the realization of the intelligent drainage at the same time, significantly reduce the cost of coal mining^9–12^. In summary, the combination of fuzzy control and Avoiding Peak Filling Valley strategy, its application in the mine drainage system, can realize the mine intelligent drainage and increase the economic benefits of the enterprise. At the same time, intelligent drainage reduces the direct participation of the personnel, and the safety of coal mine production has been dramatically strengthened, which is of great reference significance for the construction of intelligent coal mines and provides the theoretical basis for the same type of research^13–15^.

With the development of intelligent control technology and sensor technology, the mine drainage method develops in the direction of intelligence, in which fuzzy control provides a theoretical basis for the mine drainage system to realize intelligent control and many scholars have done research on such issues and achieved fruitful results. The former Soviet Union design institute^16,17^ developed a system software package according to the complex underground environment of the coal mine, the amount of water influx and mine drainage parameters, and has certain computer-aided functions which can optimize the operation of the drainage system management. This research provided a reference for a large number of subsequent studies, but with the development of technology, this system program cumbersome, low degree of automation, versatility and other shortcomings slowly exposed, no longer suitable for modern mining production. Zambia KCM company^18^ Konkola mine in the underground drainage pump room chose Rockwell A–B PLC controller design drainage system, and the United States HOMETWELL coal company developed a PLC-based mine automatic drainage safety monitoring system; this kind of research has dramatically improved the degree of automation of the drainage system, but can only be based on the information of the water level to start the pump. Hence, the degree of intelligence is low! However, it can only start the pump according to the water level information. Hence, the degree of intelligence could be higher, and it cannot take advantage of the dynamic change in electricity cost to realize resource savings. In addition, in Canada, Australia, Finland and other coal countries, advanced automation technology and sensor technology have been applied to coal drainage systems. Canadian scientists first put forward the concept of a "digital mine", which will be intelligent, uncrewed aerial vehicles, information technology and coal mine production process data collection and monitoring combined through a large number of sensors, detection and PLC automatic control to achieve many sensors. Through many sensors and PLC automatic control, real-time monitoring of mine drainage equipment and remote automation control^19–21^. Finland's Coal Mining Industry Association put forward the concept of "intelligent mine technology", mainly relying on wireless information and network technology to achieve all aspects of coal mine data collection and transmission through PLC automatic control to achieve coal mine production automation and mining real-time monitoring purposes^22^. Chinese scholar Liu Wei^23^ researches the application of the artificial group peak algorithm in pump group scheduling optimization and puts forward the pump group optimization scheduling scheme based on the artificial group peak algorithm, but does not take into account the impact of the complexity of the underground working conditions, and can not realize the on-line real-time optimization, and can not guarantee the reasonableness and timeliness of the control. Barnard et al., through the study of energy consumption of mine water pumping systems in South Africa, proposed to reduce the electricity cost of mine water pumping systems by changing the operation mode of power demand side load. This method does reduce the electricity cost, but the pumping load is also decreased at the same time, but there is a certain degree of safety hazards^24^.

Previous research has some problems, such as the drainage system control method being single, poor reliability and low efficiency; drainage work is manually completed by the site personnel, water level status, pump status, valve status, pump operation time, etc., are completed by the experience of the judgment, which is easy to lead to errors in the operation of the safety accidents caused by a large number of wasted workforce and material resources. At the same time, the existing “Avoiding Peak Filling Valley” control strategy is too simple, and most of the drainage work is carried out by the traditional high water level method, which is economically ineffective and has potential safety hazards. In this study, according to the specific parameters of the underground water storage and the rate of mine water inflow, MATLAB software is used to design a three-dimensional fuzzy control strategy^25,26^. Then, the Avoiding Peak Filling Valley strategy is introduced into the process of fuzzy control rule design to form a three-dimensional fuzzy control mechanism, i.e., three-dimensional fuzzy control of the time, level, and rate of change of the water influx, which can take into account the intelligent drainage^27,28^ and at the same time save the cost of coal mining, and solve the problems of the previous studies. After extracting the data from the mine water bin after using the unoptimized dynamic planning method, simulation experiments were conducted on the same data using the optimized dynamic planning method. It was found that the electricity cost consumed before optimization was 52,586 yuan/day, while the electricity cost consumed after optimization was reduced to 41,692 yuan/day. The electricity cost spent per day was reduced by 20.69% compared with the previous, and a year can be saved about 3,969,730 yuan, significantly reducing the production cost of coal mining enterprises.

## Research on intelligent control strategy of mine water bin drainage

### Fuzzy control theory

Fuzzy control is a nonlinear intelligent control based on fuzzy set theory, linguistic variables, and logical reasoning. The basic idea is to establish a system control strategy from the experience summarized in the long-term practice of expert technicians, transform the fuzzy control language rules into corresponding numerical operations, and use fuzzy mathematics as the theoretical basis and the computer as the medium for nonlinear intelligent control^29^.

According to the number of input variables, fuzzy controllers can be divided into one-dimensional, two-dimensional and three-dimensional fuzzy controllers. Currently, the most widely used fuzzy controller is the two-dimensional controller. A three-dimensional fuzzy controller has three input variables: system deviation E, deviation change Ec and deviation change rate of change Ecc. A three-dimensional fuzzy controller is much better than a two-dimensional controller in control effect, and it can reflect the dynamic characteristics of the output variables in the controlled process more strictly^30^.

**Figure 2:**
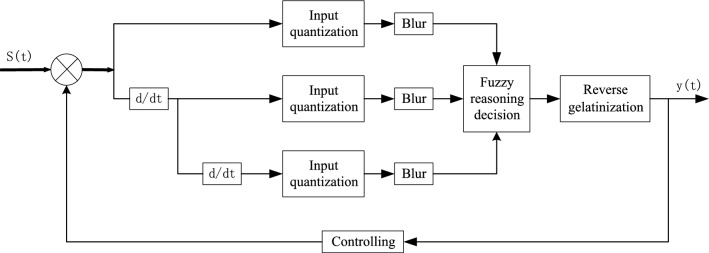
Working principle of 3D fuzzy controller.

The underground coal mine drainage program should consider the height of the water level in the water bin, the rate of change of the surge water and the period of the electricity price. Therefore, a three-input single-output fuzzy controller (three-dimensional fuzzy controller) is designed^31^, with the input variables of water level height E, gushing water change rate Ec and period Time. The output variable is the number of pumps turned on O. The three-dimensional fuzzy controller fuzzified the three input variables, and the degree of affiliation of the fuzzy set is found on the domain of the affiliation function, which is then transformed into a fuzzy variable. Fuzzy decision-making is carried out according to the fuzzy control rules. After obtaining the fuzzy control quantity, non-fuzzy processing is done to obtain the exact output variable. The working principle of a 3D fuzzy controller is shown in Fig. 2.

### Avoiding Peak Filling valley strategy

Most of the mines are often in the peak period of electricity consumption for drainage operations when the high price of electricity leads to increased cost of electricity for drainage. Given this problem, the formulation of fuzzy control rules to follow the " Avoiding Peak Filling Valley" strategy, through the water level of the water tank is subdivided. Different control logic is set for each water level interval, and at the same time, the control logic can be adjusted dynamically according to the size of the water influx. An adjustment period is set for adjusting the control logic. The control logic can be dynamically adjusted according to the size of the water influx and the adjustment period, adjust the control logic. Reduce the number of water pumps running during the peak period of electricity consumption, thus reducing the cost of electricity consumption in the drainage system. A mine in Henan Province is located in the region of the power supply industry to provide electricity price notation table as shown in Table 1.

**Table 1:** Electricity prices by time period.

Time	8:00–10:00	10:00–12:00	12:00–18:00	18:00–22:00	22:00–8:00
Electricity usage period	General period	Peak period	General period	Peak period	Low valley period
Price (yuan/kWh)	1.00	1.80	1.00	1.80	0.50

The electricity of a day is divided into general time, low time and peak time; the general time is 8:00–10:00 and 12:00–22:00; the low time is 22:00–8:00; and the peak time is 10:00–12:00 and 18:00–22:00. Based on the above scenario if the series of scheduling of the pumps is optimized, for example, to make full use of peak time If the pump scheduling series is optimized, such as making full use of the time before the arrival of the peak hours, giving full play to the water storage capacity of the water silo, not starting the pumps as much as possible during the peak hours, starting the pumps according to the needs during the general hours, and starting the pumps sufficiently during the trough hours, to meet the normal drainage of the water silo in the mines and to keep the cost of electricity low enough to achieve the maximum utilization rate of the resources.

## Fuzzy controller design

### Identification of fuzzy variables

The fuzzy control of the system incorporates a "Avoiding Peak Filling Valley " strategy. According to the different tariff periods, categorized into peak, low valley and general periods, the physical domain of the input variable time t is chosen as [0, 24]. The time variable t is divided into three fuzzy subsets: NB (Low valley period), NS (Peak period), PS (General period), and its fuzzy linguistic variable is time divided into four fuzzy theoretical domains. T = {− 1, 0, 1}^32^.

Define the lowest water level of the downhole water bin as H0, and the actual measured water level height as H. Obtain the water level deviation E = ΔH = H − H0, and choose its physical theory domain as [0, 4]. The deviation E is categorized into five fuzzy subsets: NB (low water level), NS (lower water level), ZE (middle water level), PS (higher water level), and PB (high water level). The fuzzy domains are categorized into five levels according to the range of variation of the deviation E. E = {− 2, − 1, 0, 1, 2}.

The input variable Ec represents the rate of change of water level deviation, and its physical domain is chosen as [− 0.5, 0.5]. The rate of change of water level deviation Ec is divided into five fuzzy subsets: NB (water level falling very fast), NS (water level falling slowly), ZE (water level stabilizing), PS (water level rising slowly), and PB (water level rising very fast), whose fuzzy theories domains are classified into five fuzzy levels. EC = {− 2, − 1, 0, 1, 2}.

The single output variable of the controller of this system is the number of starting units of water pumps, whose physical domain is chosen as [0, 4], and five fuzzy subsets are selected: ZERO (full stop), ONE (running one unit), TWE (running two units), THREE (running three units), FOUR (complete start). The fuzzy linguistic variable for the number of starting units of water pumps is O, which is divided into five fuzzy domains. O = {− 2, − 1, 0, 1, 2}.

### Determine the affiliation function

**Figure 3:**
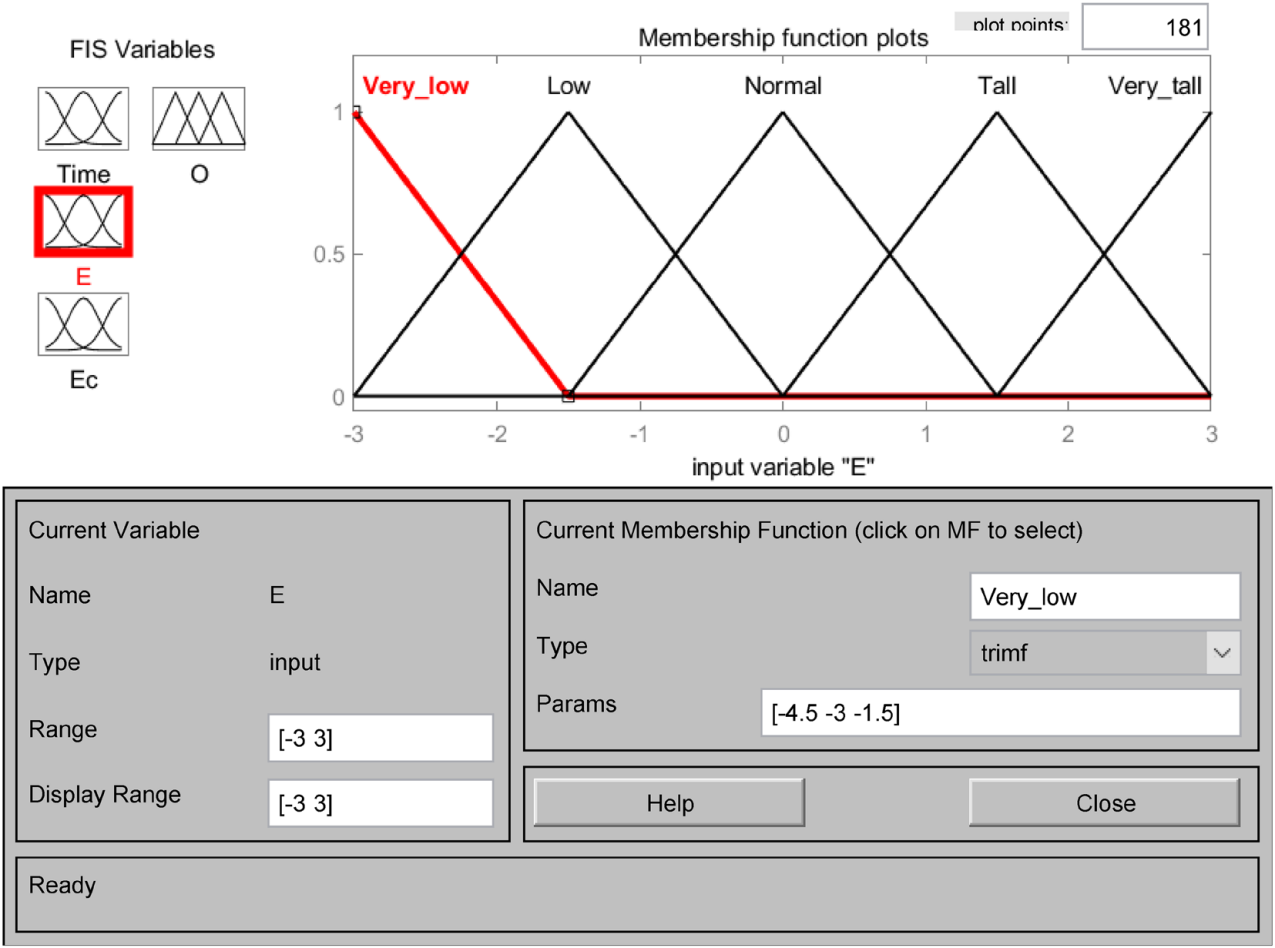
Affiliation function of E.

**Figure 4:**
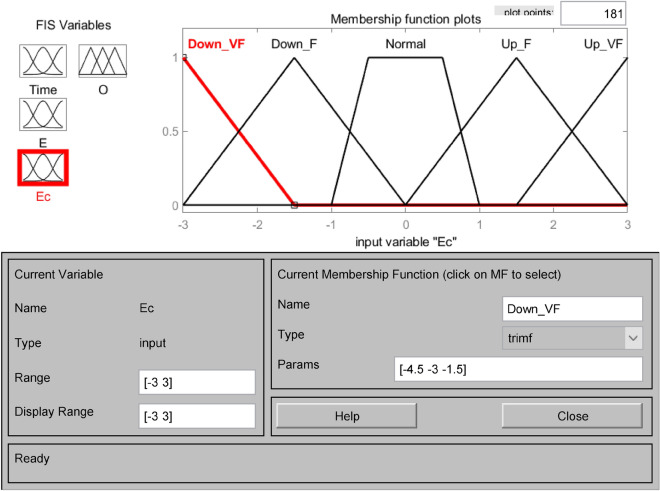
Affiliation function of Ec.

**Figure 5:**
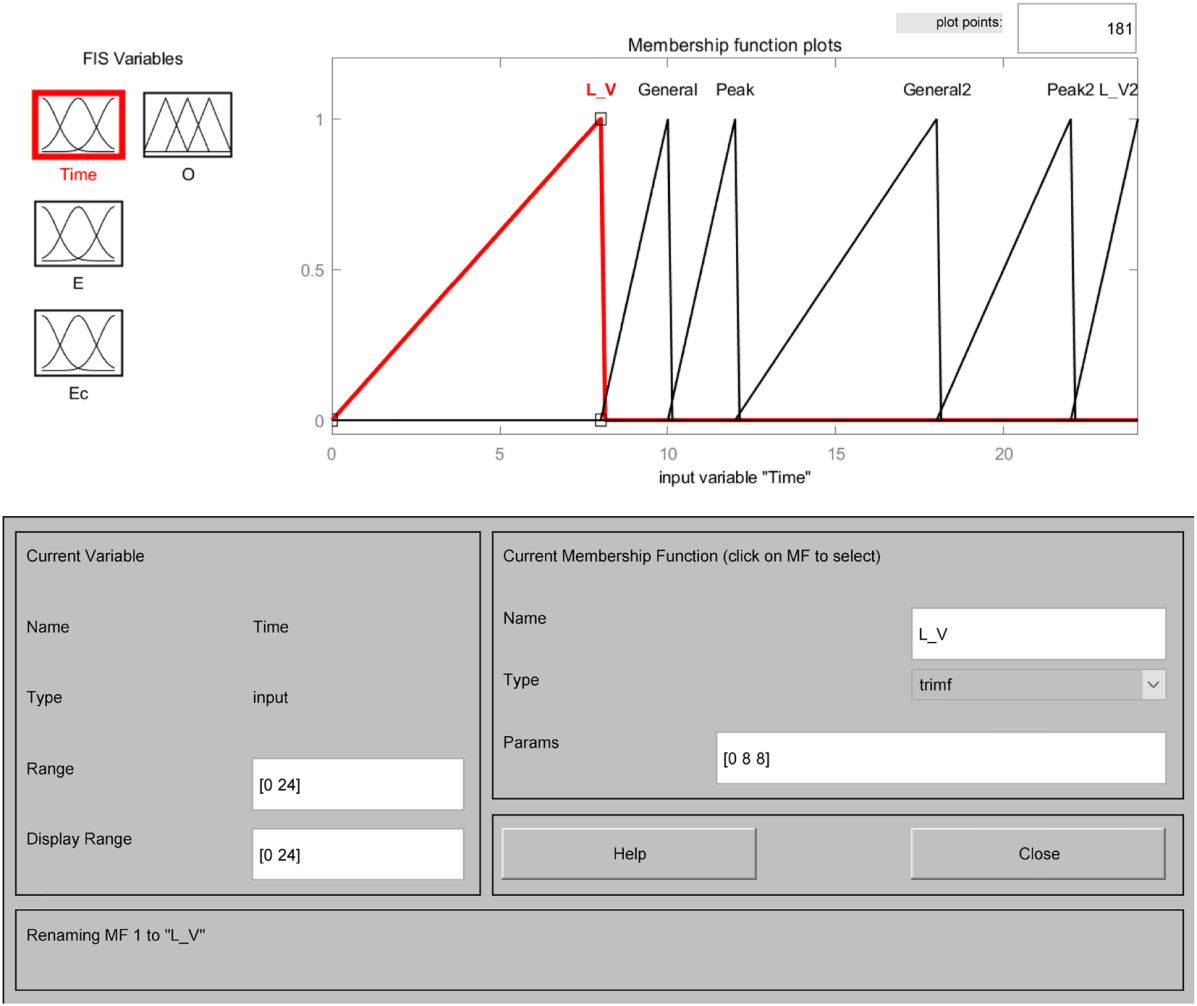
Affiliation function of Time.

**Figure 6:**
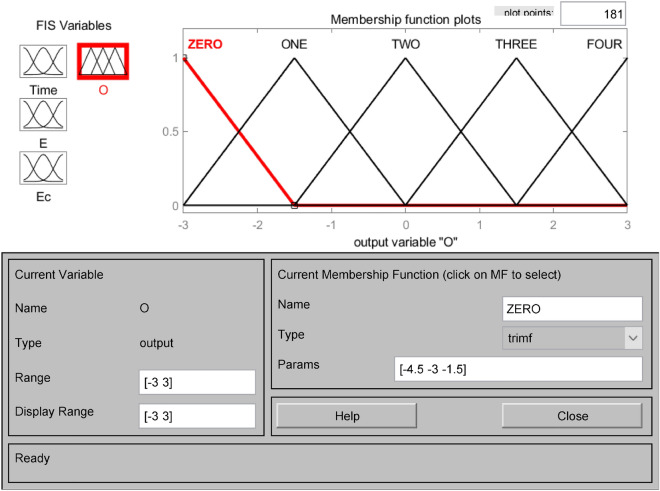
Affiliation function of O.

The affiliation functions are Gaussian, triangular, bell-shaped, S-shaped, etc., which characterize the degree to which the elements Xe in any essential thesis domain belong to the fuzzy set E. Choosing different affiliation functions will significantly affect the fuzzy control effect. When the slope of the shape of the affiliation function curve is more significant, and the distribution area is small, it reflects the higher resolution of the fuzzy set and the higher sensitivity of the control. Otherwise, the system fuzzy control effect is relatively flat and stable^33^. This system chooses the trigonometric function as the subordinate function, which has a larger sensitivity and can quickly generate the corresponding control signal. Open the FIS Editor in MATLAB and establish the subordinate functions of the input variables water level deviation E, water level deviation change rate Ec, drainage period Time, and the output variable number of pump start-up units O, respectively. As shown in Figs. 3, 4, 5 and 6. Figure 3 shows the affiliation function of E, Fig. 4 shows the affiliation function of Ec, Fig. 5 shows the affiliation function of Time and Fig. 6 shows the affiliation function of O^34^.

### Determining fuzzy control rules

**Figure 7:**
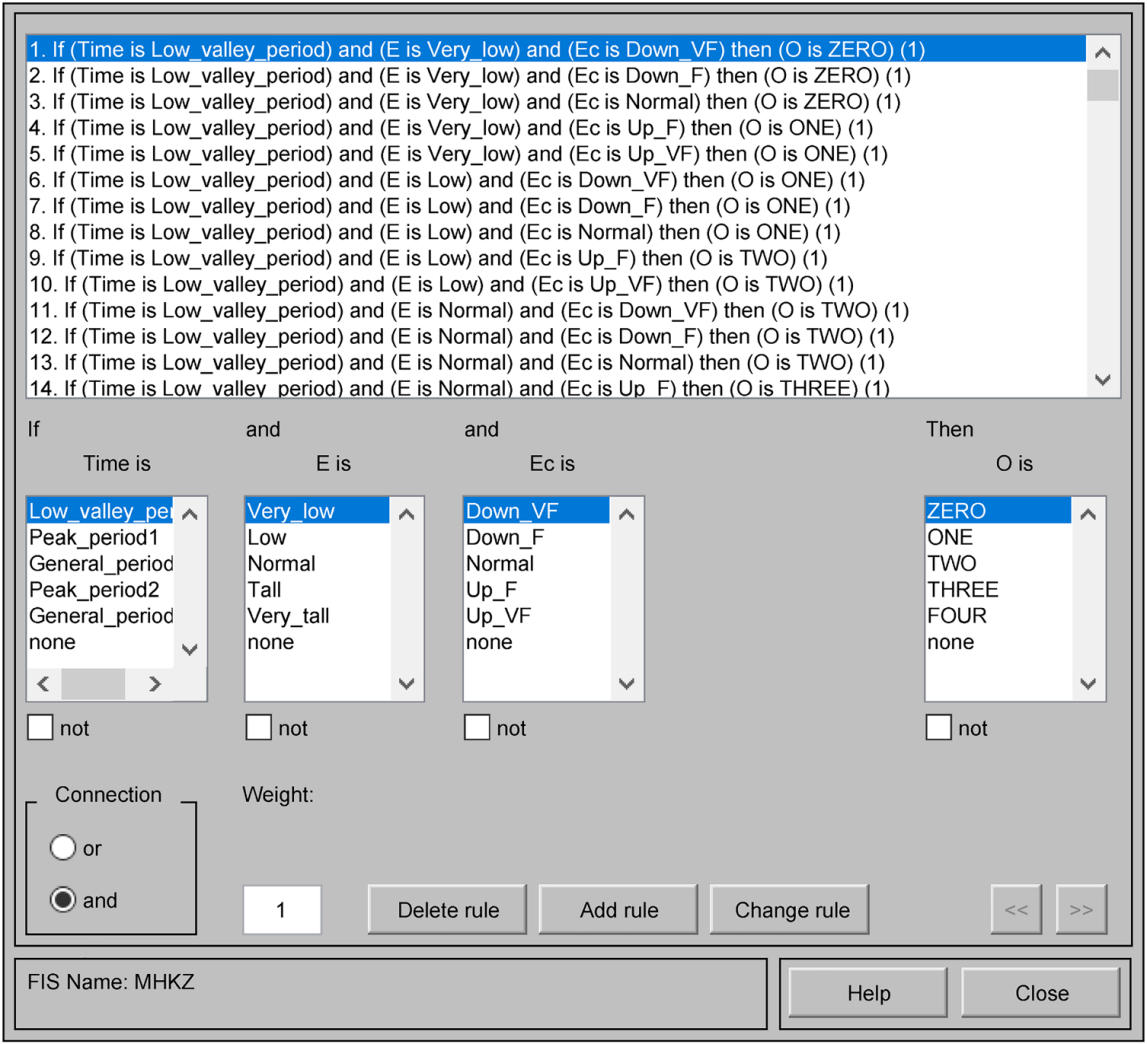
Fuzzy control rules.

**Figure 8:**
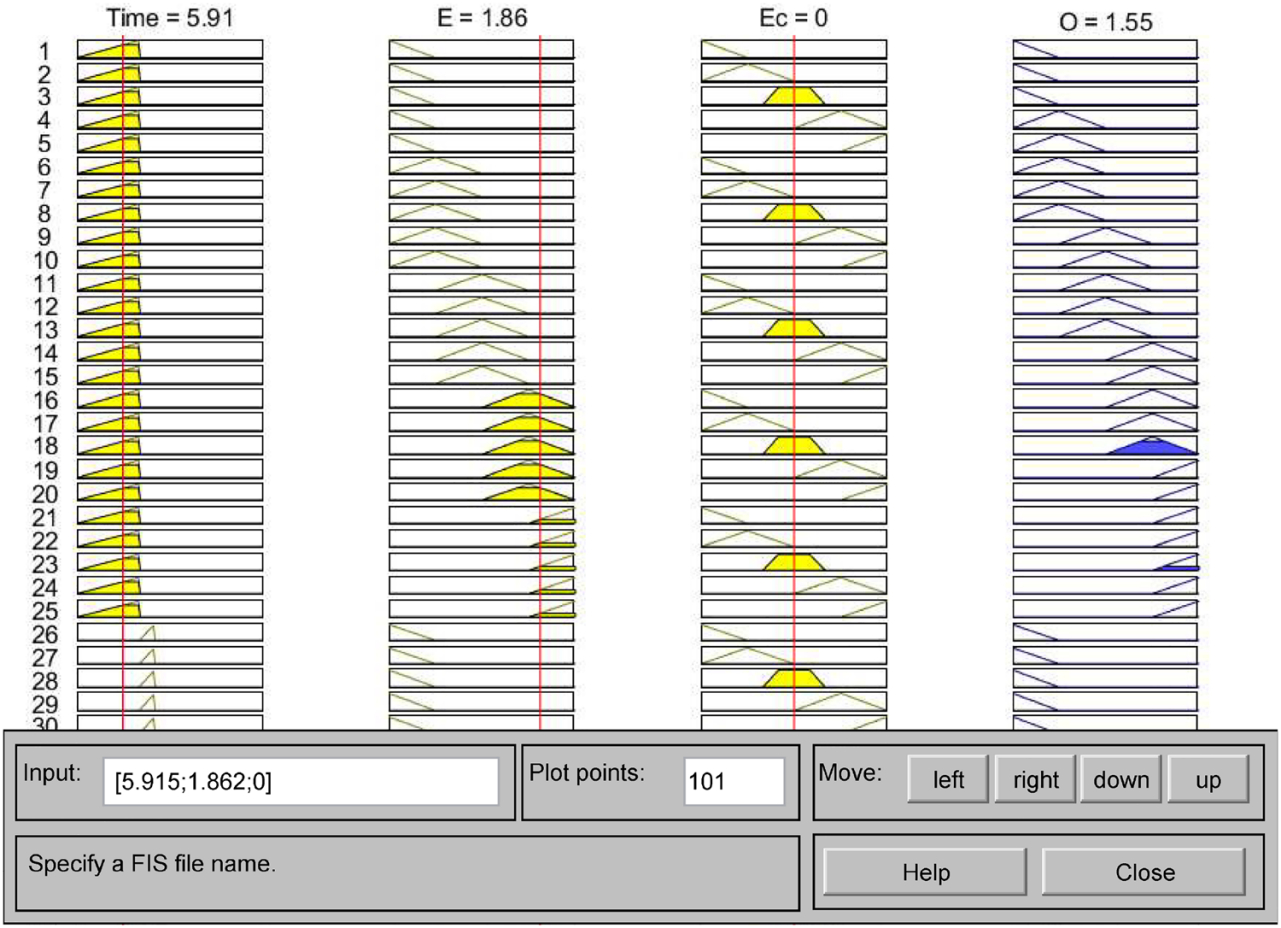
Observed fuzzy control rules.

In order to ensure the control effect and improve the arithmetic efficiency, there should not be too many fuzzy control rules^35^. The trough time and general time of the variable time t are regarded as one kind of control rule, and the peak time is regarded as one kind of control rule, so there are 50 kinds of fuzzy control rules for the mine water bin drainage system, and the control rules are shown in Table 2. The fuzzy control rules are established in the fuzzy rule editor under the MATLAB fuzzy logic toolbox and observed under the fuzzy rule observation window, as shown in Figs. 7 and 8. Figure 7 shows the fuzzy control rules, and Fig. 8 shows the observed fuzzy control rules^36^.

**Table 2:** Table of fuzzy control rules.

Period	E	Ec				
		NM	NS	ZE	PS	PM
Low valley and General period	NM	ZERO	ZERO	ZERO	ONE	ONE
	NS	ONE	ONE	ONE	TWO	TWO
	ZE	TWO	TWO	TWO	THREE	THREE
	PS	THREE	THREE	THREE	FOUR	FOUR
	PM	FOUR	FOUR	FOUR	FOUR	FOUR
Peak period	NM	ZERO	ZERO	ZERO	ZERO	ZERO
	NS	ZERO	ZERO	ZERO	ONE	ONE
	ZE	ONE	ONE	ONE	TWO	TWO
	PS	TWO	TWO	TWO	THREE	THREE
	PM	THREE	THREE	FOUR	FOUR	FOUR

**Figure 9:**
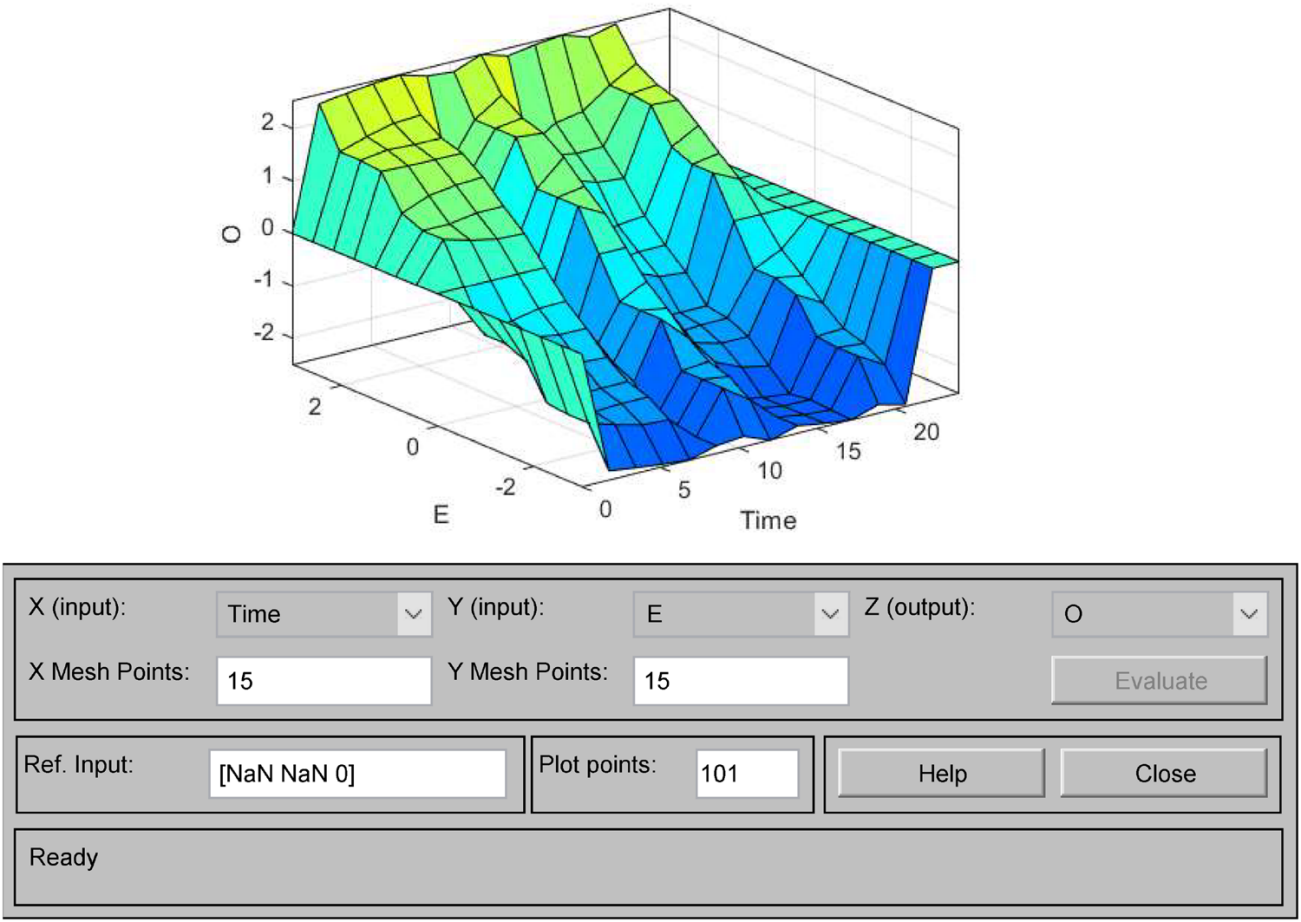
E-Time-O output surface.

**Figure 10:**
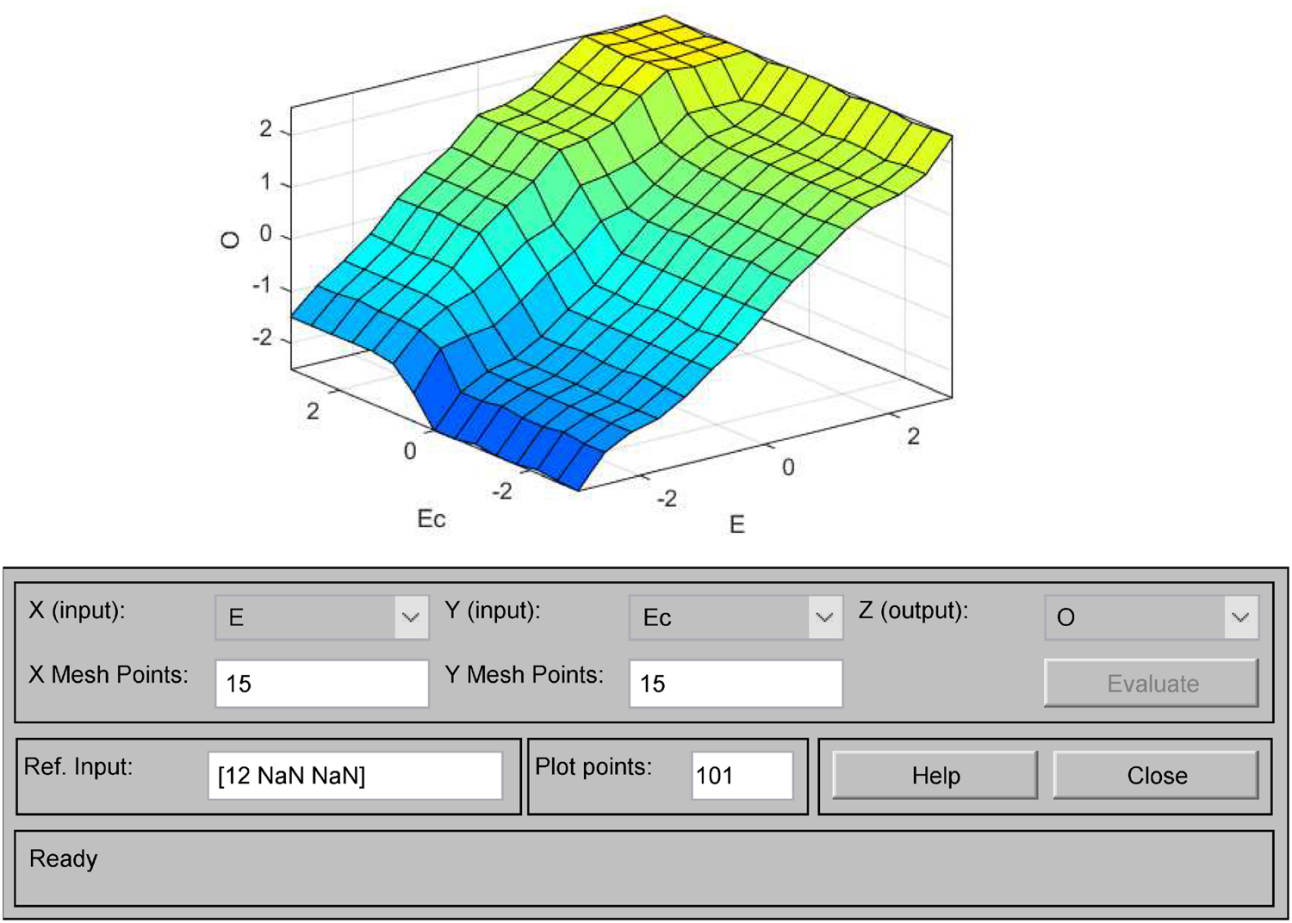
E-Ec-O output surface.

**Figure 11:**
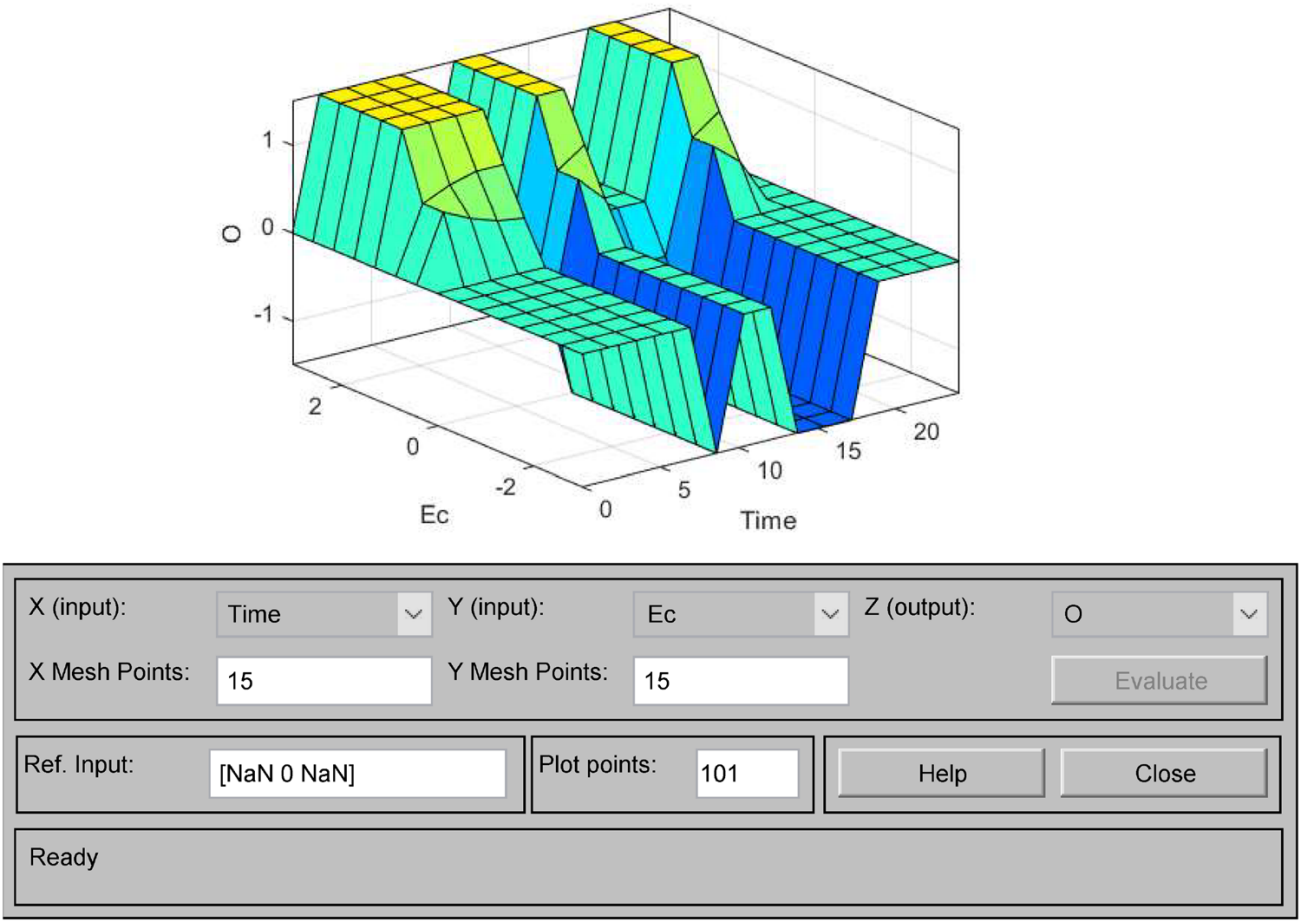
Ec-Time-O output surface.

The output surfaces of the established water level fuzzy controller are observed in the Surface Viewer of the MATLAB Fuzzy Logic Toolbox, Fig. 9 shows the E-Time-O output surface, Fig. 10 shows the E-Ec-O output surface and Fig. 11 shows the Ec-Time-O output surface.

## Experimental analysis and validation

### Experimental analysis before unoptimized dynamic programming

**Figure 12:**
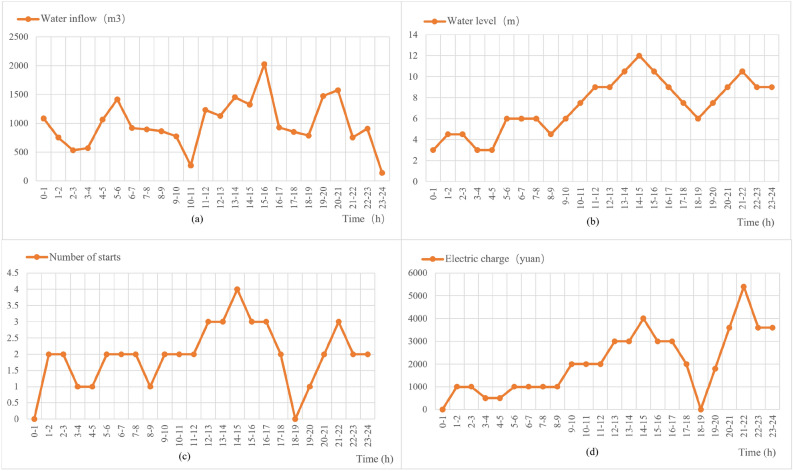
Unoptimized dynamic planning data.

In order to verify the actual effect of the designed method, the actual mine water storage surge volume of a day for comparison and verification, first of all, the real access to a day of mine water surge and the required electricity cost data, which we call the unoptimized dynamic planning before the data. Unoptimized dynamic planning before the time and water influx, time and water level, time and the number of pumps started, time and electricity cost relationship graph, respectively, as shown in Fig. 12a–d.

**Figure 13:**
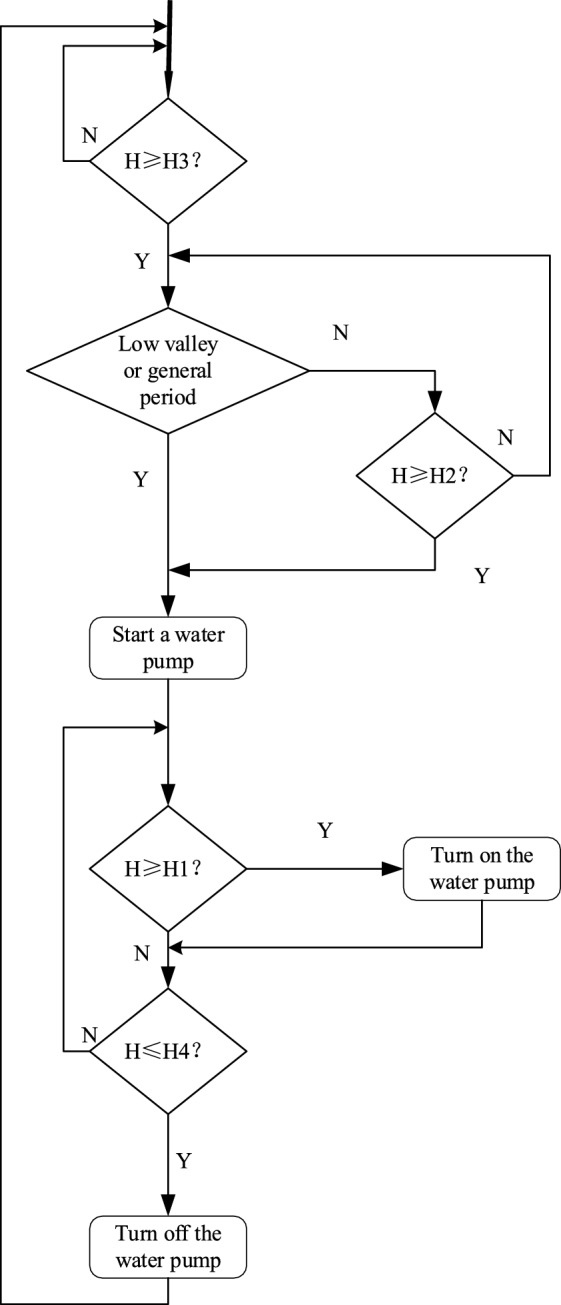
Flowchart of original peak avoidance control principles.

A drainage system before optimal dynamic planning methods^37,38^ is a relatively simple control strategy for peak avoidance and valley filling. The principle of tariff peak and valley avoidance is as follows: four different water level criteria are used, which are the limit level H1, the over-limit level H2, the alarm level H3, and the lower limit of the water level H4. When the water level reaches the alarm level H3, the grid load is detected first. The pump will be started immediately in the low or general time, and the pump will be suspended in the peak time. When the water level reaches the over-limit level of H2, the pumps will be forced to start immediately. When the water level reaches the limit level, it means that the discharge of one pump^39^ can no longer remove the mine surge water, and the second pump needs to be started. When the second pump joins, the operation is still unable to reduce the water level to the lower limit of the water level; it is necessary to continue to use the third pump, and so on. The specific original peak avoidance control principle flow chart shown in Fig. 13^40^.

### Post-experimental analysis of optimized dynamic programming

**Figure 14:**
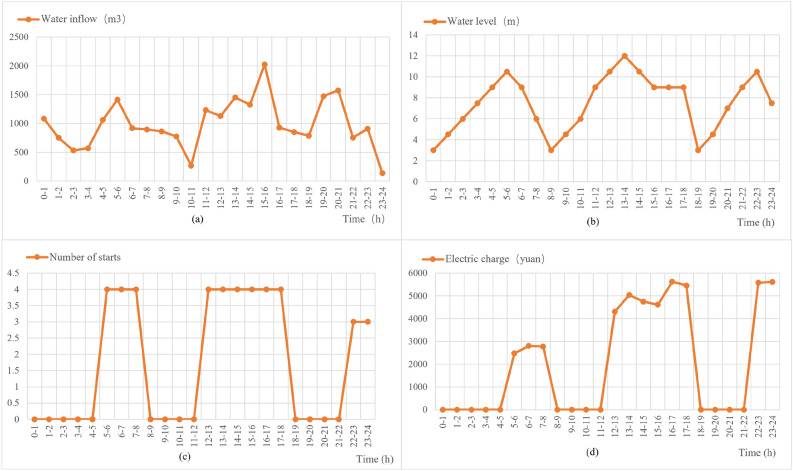
Data after optimized dynamic planning.

The unoptimized dynamic planning method specifies the number of pumps to be turned on according to the tariffs at different time stages and the silos at different water levels, but there are some problems, such as not being able to drain the water before the peak tariffs, which leads to the need to turn on the pumps during the peak tariffs, and at the same time, the control strategy before the dynamic planning does not incorporate the rate of change in the surge volume, which has a great potential for improvement in terms of reliability and economy. At the same time, the control strategy before dynamic planning cannot incorporate the rate of change of water influx, which leaves much room for improvement in terms of reliability and economy. In this regard, we use the optimized dynamic planning control strategy proposed in this paper for simulation experiments with the same influx data as the control strategy before the optimized dynamic planning. And the relationship between time and water influx, time and water level, time and number of pump starts, time and electricity cost are plotted as shown in Fig. 14a–d, respectively.

As can be seen from Figs. 13 and 14, the optimized dynamic planning strategy has a significant improvement in saving electricity cost, effectively using the water bin capacity to store mine water in the peak time of electricity cost, reducing the opportunity for pumps to turn on, and making full use of the lower electricity cost in these two periods to turn on the pumps in the general time and the low valley time, and emptying the water bin as soon as possible to prepare for the arrival of the peak time, so that most of the large-scale electricity consumption periods stayed in the tariff general hours and low valley hours.

### Comparative analysis of experimental results

**Figure 15:**
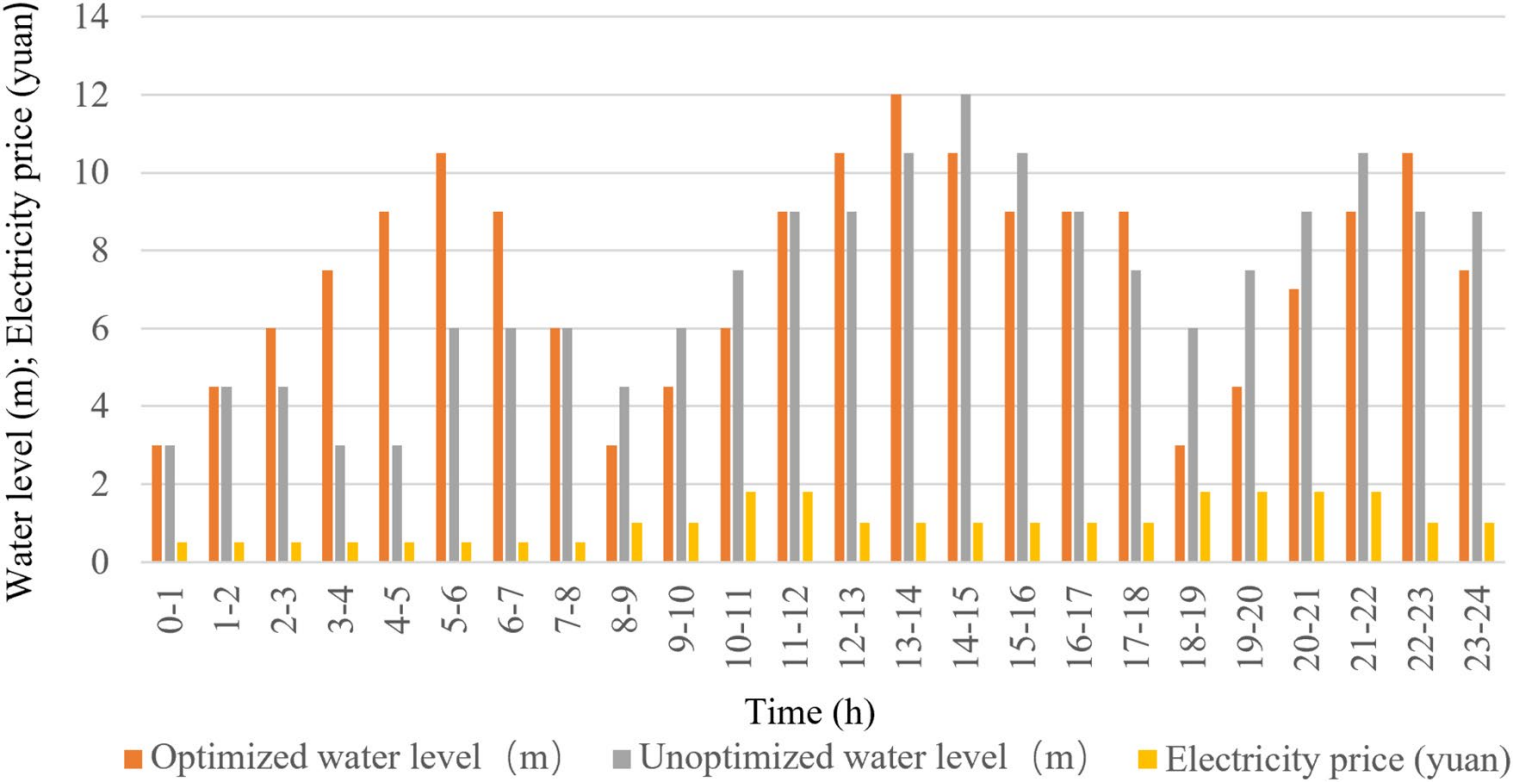
Water level comparison chart.

**Figure 16:**
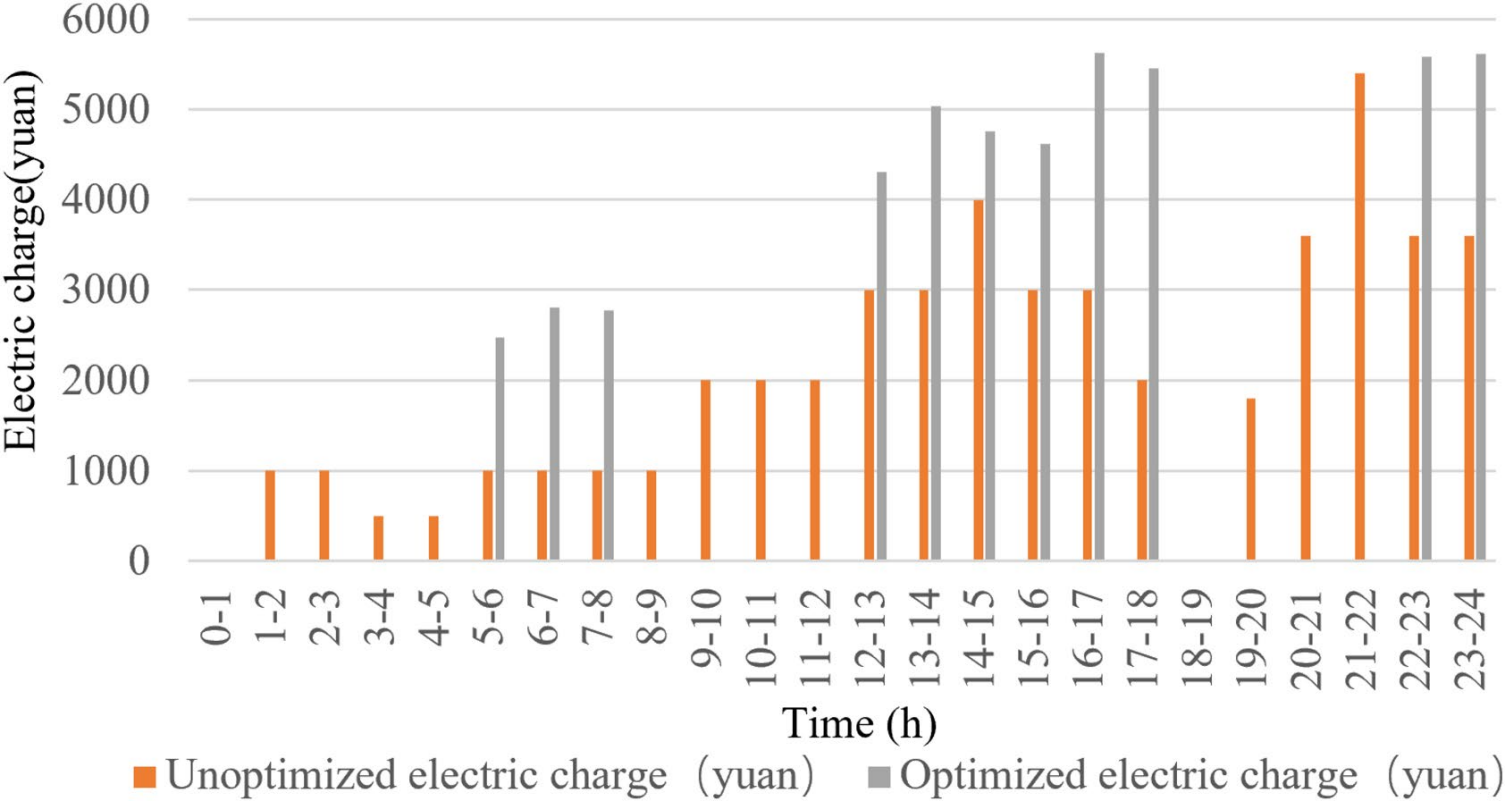
Comparison chart of electricity charge.

After optimization, the cost-saving effect of the system has been quite obvious. According to the statistical data and its analysis, the difference between the cost of electricity before optimization and after optimization is quite obvious: the cost of electricity consumed by the pumping station system before optimization is 52,586 yuan/day, while the cost of electricity consumed after optimization is reduced to 41,692 yuan/day, and the cost per day has been reduced by 20.69% compared with the previous one, which saves 3,969,730 yuan a year. The comparison of water level before and after optimization is shown in Fig. 15, and the comparison of electricity cost before and after optimization is shown in Fig. 16.

According to the two data plots shown above, the results of the whole process optimization are divided into 24 phases, i.e., for 24 h a day. Figure 15 indicates the status of water level change before and after optimization, and the initial water level is from 3 m. Figure 16 indicates the status of electricity tariff changes before and after optimization. It can be seen through the bar chart of the electricity tariff billing of the period the pumping system, after the optimization of dynamic planning, its operating condition is more in line with the principle of avoiding peaks and filling valleys. When in the peak phase of electricity prices, the number of pumps is 0, so the cost of electricity is also 0, but in this phase, the water level will continue to rise. The number of pumps is relatively high in the average tariff and the low tariff period, so the water level will continue to fall. After optimization of the system, the price of electricity before the arrival of the peak phase of the water level discharged to the lowest level. To avoid the price of electricity during the peak phase, the need to turn on the pumps is embarrassing so that the pumping system achieves the maximum extent of the "Avoiding Peak Filling Valley " purpose^41,42^.

## Conclusions and outlook

### Conclusions

1. This study has improved the traditional two-dimensional fuzzy controller (water level—period) from the original two-dimensional controller to the current three-dimensional fuzzy controller, that is, the water level—period—rate of change of gushing water, the three-dimensional fuzzy controller in the control effect is much better than the two-dimensional controller, and it can reflect the dynamic characteristics of the output variables in the controlled process more strictly. It has achieved good results in the experiments. It can provide a theoretical basis for improving the existing mine drainage method and a reference for the same type of research.
2. Although the dynamic planning using the traditional two-dimensional fuzzy control and Avoiding Peak Filling Valley strategy can realize part of the energy-saving effect, there is still much room for improvement. The optimized dynamic planning method using the three-dimensional fuzzy controller in combination with the Avoiding Peak Filling Valley strategy can make full use of the gap in the peak hour of the electricity price compared with the previous one and give full play to the storage capacity of the mine's water storage so that the pumps will stay on the peak hour of the electricity price, and then turn off during the general electricity price. The water pumps stay in the off state during the peak hours and then take full advantage of the low price of electricity to drain the water during the general and low hours, which can reduce the daily consumption by 20.69% compared with the previous one under the premise of guaranteeing the safety of the mine.

### Limitation and future research direction

(1) Every year, nearly one-fifth of the coal mine’s production input is used to pay the electricity cost of the dewatering system. The model proposed in this paper divides the day into three time periods, including the general time period, the low time period and the peak time period, according to the characteristics of the daily fluctuation of electricity cost, and on the basis of which the fuzzy control system is constructed by combining the water level deviation and the rate of change of water level deviation, which effectively reduces the cost of drainage system and improves the production efficiency of coal enterprises, and it also provides the ideas and references to solve the problem for other researches having the same problem. (2) The proposed model also has some limitations, such as the use of influx data using the mine’s influx data for one day, which is a bit idealistic to project a year’s savings, future research can be committed to the statistics of influx data with a larger time span, such as a year’s data, in order to improve the accuracy of the model’s savings expenditure. Meanwhile, in the water level deviation and the rate of change of water level deviation, because of the fluctuation of wastewater in the silo, the input of the model can be added to the filtering process of the sensor, and these fluctuations can also be filtered out by the algorithm in the model, so as to increase the accuracy of the model.

## Data Availability

The data can be requested from the corresponding authors.
